# Supplementary material for: The causal relationship between the human gut microbiota and pyogenic arthritis: a Mendelian randomization study
Source: Front Cell Infect Microbiol. 2024 Nov 26;14:1452480. doi: 10.3389/fcimb.2024.1452480 (PMC11629706; doi:10.3389/fcimb.2024.1452480)
Supplement: Supplementary file 1 [file DataSheet1.zip › mendelian test/ebi-a-GCST90027494.csv_forest.pdf]

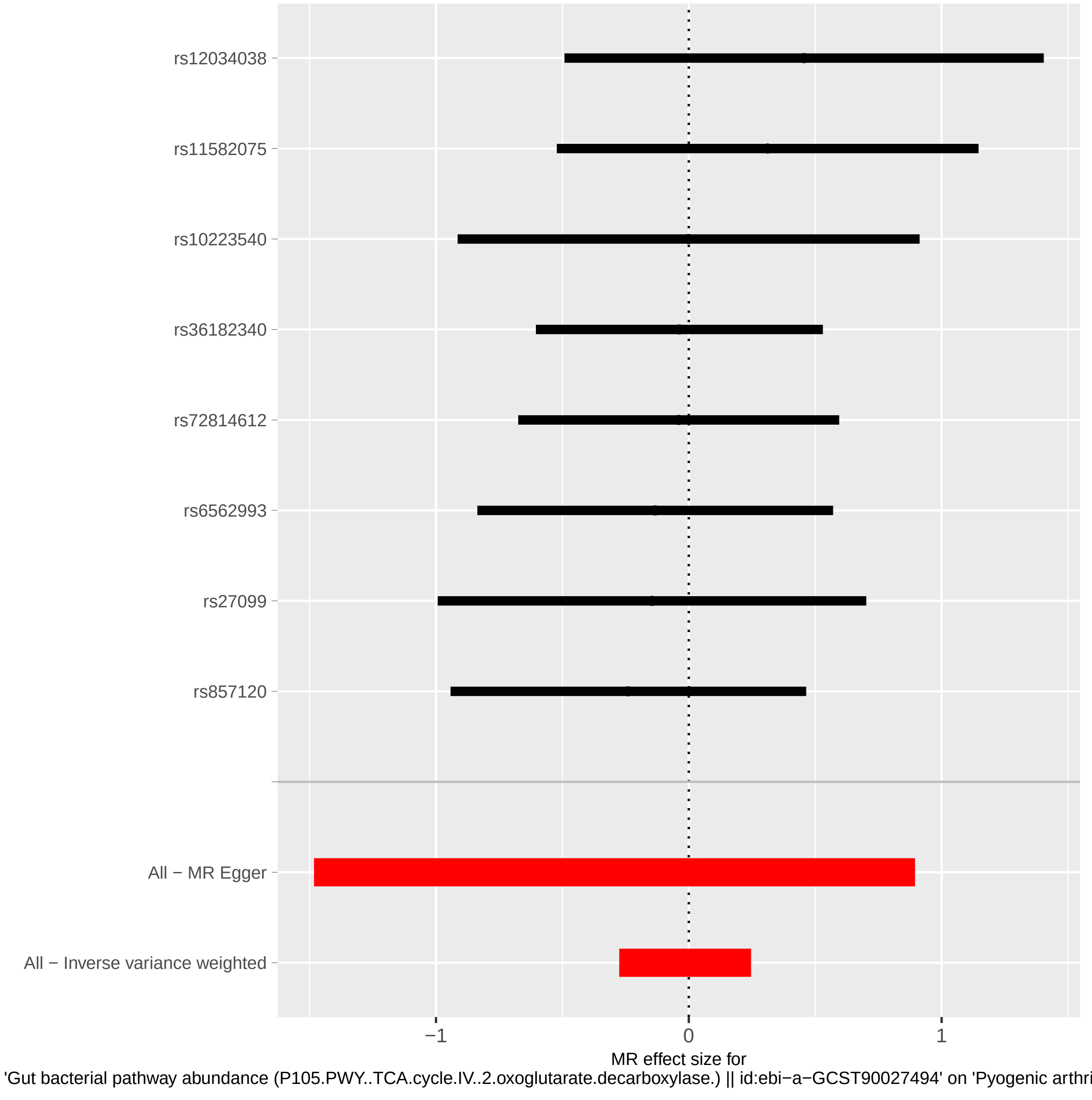
rs12034038
rs11582075
rs10223540
rs36182340
rs72814612
rs6562993
rs27099
rs857120
All − MR Egger
All − Inverse variance weighted
−1
0
1
MR effect size for
'Gut bacterial pathway abundance (P105.PWY..TCA.cycle.IV..2.oxoglutarate.decarboxylase.) || id:ebi−a−GCST90027494' on 'Pyogenic arthri
